# Influence of transition metal doping on the structural, optical, and magnetic properties of TiO_2_ films deposited on Si substrates by a sol–gel process

**DOI:** 10.1186/1556-276X-8-533

**Published:** 2013-12-19

**Authors:** Jianjun Tian, Huiping Gao, Hui Kong, Pingxiong Yang, Weifeng Zhang, Junhao Chu

**Affiliations:** 1 School of Physics and Electronics, Henan University, Kaifeng 475004, China; 2 Key Laboratory of Polar Materials and Devices, Ministry of Education, East China Normal University, 500 Dongchuan Road, Shanghai 200241, China

**Keywords:** TiO_2_, Diluted magnetic semiconductors, Phase transformation, Optical property, Magnetic property, 81.40.Ef, 74.62.Dh, 64.70.Nd, 78.20.Ci

## Abstract

Transition metal (TM)-doped TiO_2_ films (TM = Co, Ni, and Fe) were deposited on Si(100) substrates by a sol–gel method. With the same dopant content, Co dopants catalyze the anatase-to-rutile transformation (ART) more obviously than Ni and Fe doping. This is attributed to the different strain energy induced by the different dopants. The optical properties of TM-doped TiO_2_ films were studied with spectroscopic ellipsometry data. With increasing dopant content, the optical band gap (*E*_OBG_) shifts to lower energy. With the same dopant content, the *E*_OBG_ of Co-doped TiO_2_ film is the smallest and that of Fe-doped TiO_2_ film is the largest. The results are related to electric disorder due to the ART. Ferromagnetic behaviors were clearly observed for TM-doped TiO_2_ films except the undoped TiO_2_ film which is weakly magnetic. Additionally, it is found that the magnetizations of the TM-doped TiO_2_ films decrease with increasing dopant content.

## Background

Magnetic-ion-doped TiO_2_ with room-temperature ferromagnetism is one kind of promising diluted magnetic semiconductors (DMS). It has been widely studied due to its potential applications in spintronics [1-3]. Many efforts have been made to understand the mechanism of ferromagnetism (FM) in magnetic-ion-doped TiO_2_. The most important point for industrial applications is if such room-temperature FM could originate from the doped matrices and not from the dopant clusters. Some theory models, such as the Ruderman-Kittel-Kasuya-Yosida exchange [4], super exchange [5], double exchange [6], magnetic polarons [7], and *F*-center exchange mechanism [8], have been used to explain ferromagnetism in transition-metal-element-doped TiO_2_. However, many controversies still exist in the magnetic origin of DMS. Recently, room-temperature FM [9] and reversible FM [10] in undoped TiO_2_ films, and reversible FM in transition metal-doped TiO_2_ nanocrystals [11], have been reported. These reports suggest that the structural defects can induce FM order, which brings new challenges in elucidating the magnetic mechanism in this kind of DMS.

In recent years, mixed crystal TiO_2_ containing anatase and rutile phases has been more attractive because the anatase-rutile-phase junction (ARJ, related to phase composition) in the mixed crystal TiO_2_ improves the spatial charge separation and enhances the photocatalytic activity [12-16]. Disorders in the mixed crystal TiO_2_ affect the optical properties of TiO_2_[17,18]. The existence of the ARJs could enhance the disorders in the TiO_2_ films, which will change the samples' physical properties. Our recent work indicates that both doping and phase composition affect the optical properties of TiO_2_ films [19]. The ARJs could affect not only the optical but also the magnetic properties of the TiO_2_ films [20]. However, to the best of our knowledge, the effects of phase composition on the magnetic properties of doped TiO_2_ films have seldom been reported. Recently, Bahadur et al. found that the magnetic moment of the Ni-doped mixed crystalline TiO_2_ powders increases and then decreases with increasing Ni content due to the change of spin ordering [21]. However, the influence of phase composition on the magnetic properties has not been taken into account in their studies.

In this paper, transition metal (TM)-doped TiO_2_ films (TM = Co, Ni, and Fe) were deposited on Si(100) substrates by a sol–gel method. The influence of Co, Ni, and Fe doping on the crystalline structure of the TiO_2_ films was compared. The magnetic and optical properties of the TM-doped TiO_2_ films were investigated. The correlation between phase composition and magnetic and optical properties was studied, and the possible mechanism was discussed. These results will be useful for understanding the magnetic origin of oxide DMS.

## Methods

Synthesis of TM-doped TiO_2_ films, Ti_1 − *x*_TM_*x*_O_2_ (TM = Co, Ni, and Fe; *x* = 0, 0.01, and 0.03), was achieved on Si(100) substrates by sol–gel method. The precursor solutions of the TM-doped TiO_2_ films were obtained from tetrabutyl titanate, cobaltous acetate, nickel acetate, and ferric nitrate with ethanol and acetylacetone as the solvent and the chemical modifier, respectively. The details of the preparation procedure are reported elsewhere [22]. For example, to prepare a Ni-doped TiO_2_ solution, analytically pure nickel acetate (Ni[CH_3_COO]_2_) and titanium butoxide (Ti[O(CH_2_)_3_CH_3_]_4_) were used as the starting materials. Ni doping was achieved by dissolving nickel acetate in a solution with an appropriate volume ratio of ethanol (CH_3_CH_2_OH)/acetic acid (CH_3_COOH) at 60°C. Titanium butoxide and an equal amount of acetylacetone (CH_3_COCH_2_COCH_3_) were dissolved in ethanol at 30°C. Then the two solutions were mixed slowly together at room temperature. In order to get a homogenous precursor, the mixture was stirred drastically in the magnetic stirrer for 2 h at 50°C. Finally, the 0.3 mol/L precursor solution was acquired and became transparent without precipitation even after 4 months.

The silicon substrates were cleaned in an ultrasonic bath for 20 min using acetone (CH_3_COCH_3_), ethanol, and deionized water, respectively. The Ti_1 − *x*_TM_*x*_O_2_ thin films were deposited by spin coating the precursors on the silicon substrates at a speed of 3,500 rpm for 20 s. The films were thermally treated in a rapid thermal processor in air. Each layer of the films was initially dried at 200°C at a ramp rate of 15°C/s to evaporate the solvent and then rapidly heated to 380°C at a ramp rate of 20°C/s to remove the residual organics. Finally, the films were annealed at 700°C at a ramp rate of 20°C/s and naturally cooled down to room temperature. The each of the three steps of the rapid thermal treatment was held for 180 s. The spin coating and thermal treatments were repeated six times to prepare the samples.

The valences of the doping ions were determined by x-ray photoelectron spectroscopy (XPS, PHI 550 ESCA/SAM; PerkinElmer Inc., Waltham, MA, USA) with a monochromatized AlKα radiation source (*hυ* = 1,486.6 eV) operated at 10 kV and 30 mA. The electron energy analyzer was operated at the constant pass energy of 50 eV. The structures of the samples were characterized by x-ray diffraction (XRD; D/max2200VPC, Rigaku Co., Shibuya-Ku, Tokyo, Japan) using CuK_α_ radiation (*λ* = 0.15471 nm) with a resolution of 0.04° and the *2θ* range from 10° to 65°. The ellipsometric measurements were carried out by a near-infrared to ultraviolet (NIR-UV) spectroscopy ellipsometry (SE) in the wavelength range of 300 to 826 nm (1.5 to 4.1 eV) with a spectral resolution of 2 nm (SC630UVN; Shanghai Sanco Instrument, Co., Ltd., Xuhui, Shanghai, China). The incident angle for films was 70° corresponding to the experimental optimization near the Brewster angle of the Si(100) substrates. Magnetic measurements were performed at 300 K using a vibrating sample magnetometer (PPMS-9 Quantum Design, San Diego, CA, USA), and the measured sample size is about 2 mm × 10 mm. All measurements were performed at room temperature.

## Results and discussion

### XPS of the TM-doped TiO_2_ films

**Figure 1:**
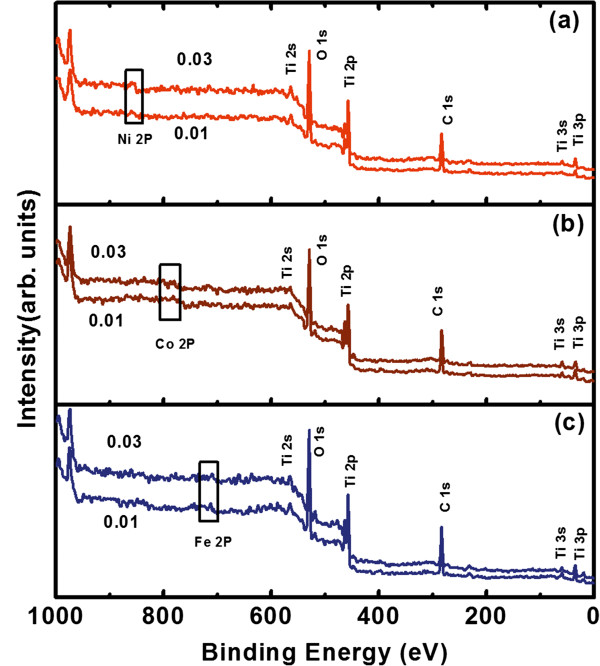
**XPS survey spectra of TM-doped TiO**_**2 **_**thin films. (a)** Ni-doped TiO_2_. **(b)** Co-doped TiO_2_. **(c)** Fe-doped TiO_2_.

**Figure 2:**
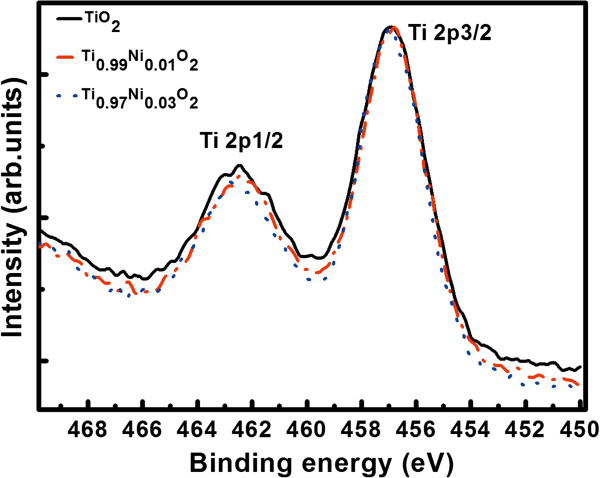
**Normalized XPS spectra of Ni-doped TiO**_**2 **_**thin films: Ti 2** ***p *** **core levels.**

Figure 1 shows the XPS survey spectra of the TM-doped TiO_2_ thin films. The carbon peak comes from surface contamination because of exposure to air [23]. All the peaks are calibrated with the carbon 1 *s* peak at 284.6 eV. The survey indicates that titanium, oxygen, iron, cobalt, and nickel are the major components on the surface of these films. Figure 2 shows a high-resolution XPS spectrum of the Ti 2*p* region for Ni-doped TiO_2_ thin films, respectively. The core level binding energy of Ti 2*p*_3/2_ is 458.4 eV and that of Ti 2*p*_1/2_ is 464.16 eV. The difference of 5.7 eV in the two peaks indicates a valence state of +4 for Ti in the TiO_2_- and Ni-doped TiO_2_ samples [24,25]. The same analysis also shows a valence state of +4 for Ti in the Fe- and Co-doped TiO_2_ samples (not shown).

**Figure 3:**
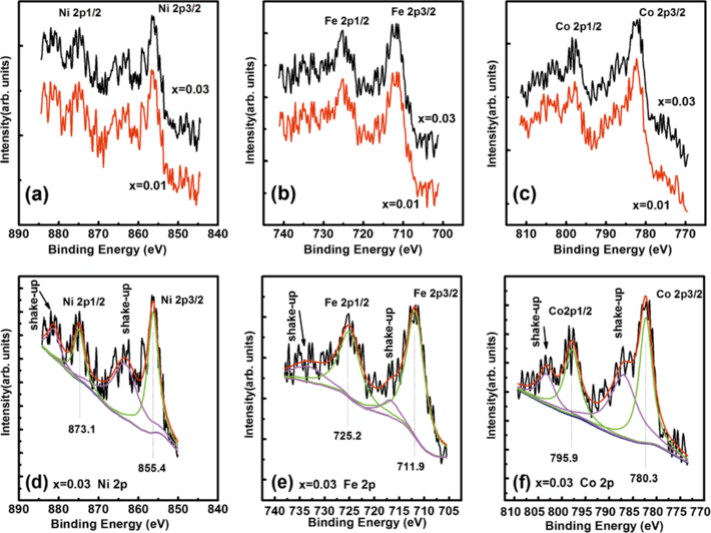
**TM 2p core level XPS spectra for TM-doped TiO**_**2 **_**thin films.** High-resolution XPS spectra of Ni 2*p***(a)**, Fe 2*p***(b)**, and Co 2*p***(c)** core level for TM-doped TiO_2_ films. Experimental and fitted XPS spectra of Ni 2*p***(d)**, Fe 2*p***(e)**, and Co 2*p***(f)** core level for Ti_0.97_TM_0.03_O_2_ films.

Figure 3 depicts the TM 2*p* core level XPS spectra for TM-doped TiO_2_ thin films. A Gaussian (80%) + Lorentzian (20%) fit was carried out and showed that the binding energy of Ni 2*p*_1/2_ is 873.1 eV; the binding energy of Ni 2*p*_3/2_ is 855.4 eV. This is different from those of metal Ni^0^ (852.6 eV) and Ni^3+^ (856.1 eV) [25,26] and very near to that of Ni^2+^ (855 eV) [21,25,27]. This indicates that the chemical valence of Ni in the films is +2. Furthermore, the difference of 17.7 eV between Ni 2*p*_3/2_ and Ni 2*p*_1/2_ peaks also indicates a valence state of +2 for Ni in the Ni-doped TiO_2_ films [25]. The same analysis also shows a valence state of +2 for Co in Co-doped TiO_2_ and a valence state of +3 for Fe in Fe-doped TiO_2_ (in Figure 3).

**Figure 4:**
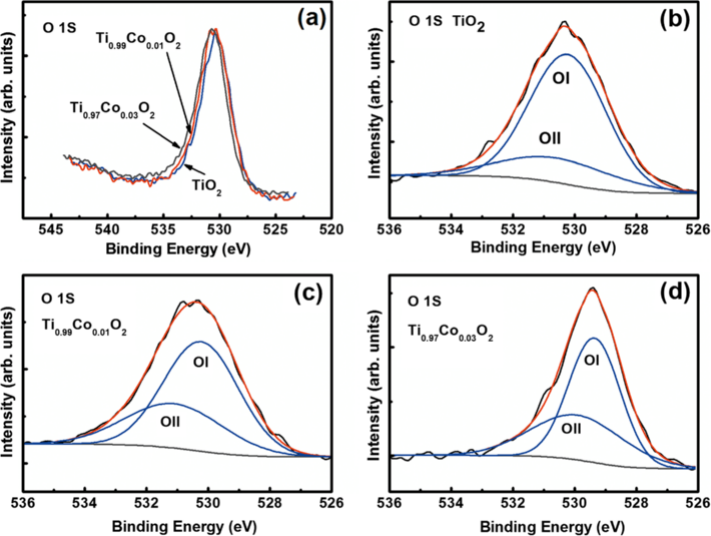
**Normalized and fitted XPS core level spectra of oxygen 1** ***s *level.** Normalized XPS core level spectra of oxygen 1 *s* level of undoped and Co-doped TiO_2_**(a)**. Fitted XPS core level spectra of oxygen 1 *s* level of TiO_2_ film **(b)**, Ti0._99_Co_0.01_O_2_ film **(c)**, and Ti_0.97_Co_0.03_O_2_ film **(d)**.

Further, TM doping may also result in oxygen vacancy due to the replacement of Ti^4+^ by TM ions to maintain crystal charge neutrality, and the vacancy content may increase with increasing dopant content. As an example, the O 1 *s* peaks for TiO_2_, Ti_0.90_Co_0.01_O_2_, and Ti_0.97_Co_0.03_O_2_ thin films are shown in Figure 4a. Both the O 1 *s* core levels display an asymmetric shape and are located at about 530.4 eV. The O 1 *s* peak was fitted by the two-peak Gaussian curves. The two fitting peaks are defined as OI and OII, respectively (Figure 4b,c,d). The OI peak is due to the oxygen atoms of TiO_2_[24,28], and the OII peak is attributed to the oxygen vacancies [24,26,29]. The OII peak appears as a function of oxygen vacancies. The increase in the area ratio of OII peak to OI peak indicates the enhancement of oxygen vacancy content [24,29,30]. The area ratio is 0.18, 0.28, and 0.32 for TiO_2_, Ti_0.90_Co_0.01_O_2_, and Ti_0.97_Co_0.03_O_2_ films, respectively. These results indicate that the oxygen vacancies increase with increasing Co content. The same analysis also suggests that oxygen vacancies increase with increasing dopant content for Fe- and Ni-doped TiO_2_ samples (not shown).

### XRD of the TM-doped TiO_2_ films

**Figure 5:**
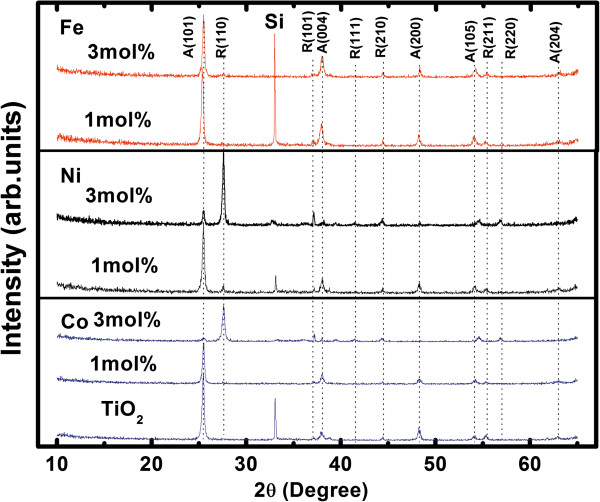
**XRD patterns of undoped and TM-doped TiO**_**2 **_**films.**

**Figure 6:**
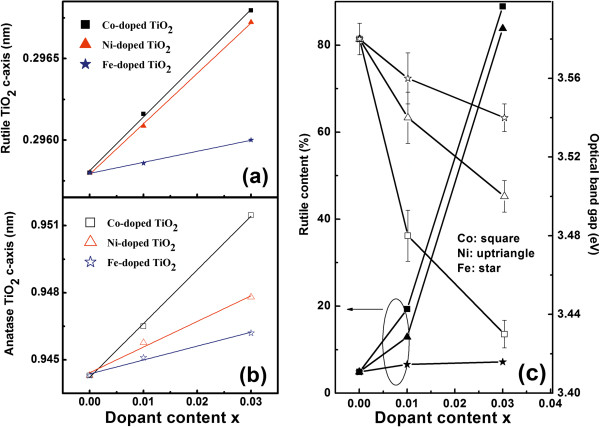
**Change in the rutile and anatase lattice constant and rutile fraction. (a,b)** The rutile/anatase TiO_2_*c*-axis length changed monotonously with increasing TM content following Vegard's law. The solid lines are the linear fitting results to guide the eyes. **(c)** Fractions of the rutile content as a function of dopant content for the TM-doped TiO_2_ films (left); evolution of the optical band gap of TM-doped TiO_2_ films with dopant content with error bar (right).

The XRD patterns of the TM-doped TiO_2_ films on silicon substrates are shown in Figure 5. All the films are mixed crystal with diffraction peaks of A(101) and R(110), respectively [20,21]. Except the diffraction peaks of the anatase and rutile phase, no impurity phase is observed, which indicates that the TM atoms have been successfully incorporated into the TiO_2_ matrix. The change in the rutile and anatase lattice constant was shown to follow Vegard's law (Figure 6a,b respectively), in which a linear relation exists between the crystal lattice constant of a material and the concentrations of the constituent elements at constant temperature [31]. Of course, TM ions may also be at the interstitial site, but the matrix compound, TiO_2_, has a relatively close-packed structure, and this is not generally favorable for interstitial defects [32]. Moreover, the interstitial defect in this case is highly charged, which is another detrimental factor [32].

With increasing dopant content, rutile-related peaks gradually increased. For the Co- and Ni-doped TiO_2_ films, when dopant content reaches 0.03, the diffraction patterns of the rutile phase become predominant. On the contrary, for the Fe-doped TiO_2_ films, the diffraction patterns of the anatase phase are still dominant. These results indicate that the addition of dopant catalyzes the anatase-to-rutile transformation (ART), which are similar to those of the Co-doped [23,33], Ni-doped [34,35], and Fe-doped [36-39] TiO_2_ powders.

The fraction of rutile phase in these films can be estimated from the XRD peak intensities by the following equation: *X*_R_ = 1/[1 + 0.884(*I*_A_/*I*_R_)], where *X*_R_ is the weight fraction of rutile phase in the samples, and *I*_A_ and *I*_R_ are the x-ray-integrated intensities of the A(101) and R(110) peaks, respectively [20]. The rutile fraction against dopant content of the TM-doped TiO_2_ films is presented in Figure 6c. It can be seen that the contents of the rutile phase enhance with increasing dopant content. The influence of the Co and Ni dopants on the ART of the TiO_2_ films is conspicuous, but minimal for the Fe dopant. At the same dopant content, the rutile content of the Co-doped TiO_2_ films is the highest, and that of Fe-doped TiO_2_ films is the lowest.

The ART is a nucleation and growth process at the expense of consuming the surrounding anatase in undoped TiO_2_[23,33]. The nuclei were formed at the anatase {112} twin boundaries. Half of the titanium cations in the twin slab displace and the rutile phase nucleates [40,41]. The transformation of bulk anatase ruptures 7 out of the 24 Ti-O bonds per unit cell and leads to the cooperative displacement of both Ti and O. After Ti^4+^ is replaced by Co^2+^, Ni^2+^, and Fe^3+^ ions, oxygen vacancies are introduced to keep the crystal charge neutrality. During the course of the ART, the presence of oxygen vacancies makes the number of Ti-O bond rupture less than 7/24 per anatase unit cell. In other words, oxygen vacancies make the ART [24].

The replacement of Ti^4+^ by Fe^3+^ leads to the following equation [32]:

(1) Fe2O3TiO22FeTi'+VO..+3OO

Similarly, the replacement of Ti^4+^ by Co^2+^ (Ni^2+^) leads to the following equation:

(2) CoOTiO2CoTi'+Vo..+Oo

Therefore, at the same dopant content, the oxygen vacancies due to Co^2+^ or Ni^2+^ doping are theoretically more than those of Fe^3+^ doping. Thus, the rutile content of Co- or Ni-doped TiO_2_ films is more than that of the Fe-doped TiO_2_ films. In addition, the ionic radius of Co^2+^, Ni^2+^, Fe^3+^, and Ti^4+^ are 0.72, 0.69, 0.64, and 0.605 Å, respectively. When the Ti^4+^ ions are substituted by TM^*n*+^ (Co^2+^, Ni^2+^, and Fe^3+^) ions, the difference in ionic radii between Ti^4+^ and TM^*n*+^ results in the lattice deformation of anatase TiO_2_, and the strain energy due to the lattice deformation facilitates the ART [33]. Furthermore, the strain energy supplied by Co^2+^ doping is bigger than that of Ni^2+^ doping because the ionic radii of Co^2+^ is larger than that of Ni^2+^. Thus, the rutile content of Co-doped TiO_2_ films is more than that of Ni-doped TiO_2_ films.

### Ellipsometric spectra of the TM-doped TiO_2_ films

With increasing dopant content, the optical properties of the doped TiO_2_ films will change due to the increasing rutile content. SE is an appropriate tool to calculate optical constants/dielectric functions and the thickness of films because of its sensitivity and nondestructivity. The SE parameters *Ψ*(*E*) and *Δ*(*E*) are the functions of the incident angle, optical constants, and the film thickness. In our previous studies, the optical constants of some materials have been successfully obtained using the SE technique [42,43]. To estimate the optical constants/dielectric functions of TM-doped TiO_2_ films, a four-phase layered system (air/surface rough layer/film/substrate, all assumed to be optically isotropic) [43] was utilized to study the SE spectra. A Bruggeman effective medium approximation is used to calculate the effective dielectric function of the rough layer that is assumed to consist of 50% TiO_2_ and 50% voids of refractive index unity [43]. Considering the contribution of the M_0_-type critical point with the lowest three dimensions, its dielectric function can be calculated by Adachi's model: *ϵ*(*Ε*) = ϵ_*∞*_ + {*A*_0_[2 − (1 + *χ*_0_)^1/2^ − (1 − *χ*_0_)^1/2^]}/(*E*_*OBG*_^2/3^*χ*_0_^2^), where, *E* is the incident photon energy, *ϵ*_*∞*_ is the high-frequency dielectric constant, *χ*_0_ = (*E* + *iΓ*), *E*_OBG_ is the optical gap energy, and *A*_0_ and *Γ* are the strength and broadening parameters of the *E*_OBG_ transition, respectively [42,44].

**Figure 7:**
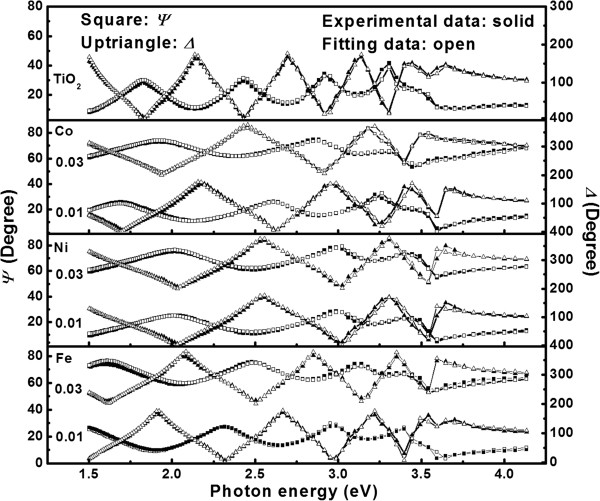
**Experimental and simulated SE of undoped and TM-doped TiO**_**2 **_**films at incident angle 70.** For clarity, each spectrum of Δ and *Ψ* are shifted by 200° and 50°, respectively.

Figure 7 shows the measured SE parameters *Ψ*(*E*) and *Δ*(*E*) spectra at the incident angle of 70° for the TM-doped TiO_2_ films on Si substrates. The Fabry-Pérot interference oscillations due to multiple reflections within the film have been found in from 1.5 to 3.5 eV (354 to 826 nm) [42,43]. Note that the interference oscillation period is similar across the film samples, except for the undoped TiO_2_ that has the maximum thickness. The revised Levenberg-Marquardt algorithm in the nonlinear least squares curve fitting can extract the best-fit parameter values in the Adachi's model for all samples. The simulated data are also shown in Figure 7. The good agreement between experimental and fitting curves suggests that the optical constants attained by the simulation are reliable.

The fitted parameters of the TM-doped TiO_2_ films determined by the SE spectra are listed in Table 1. From the table, the film thickness of undoped TiO_2_ film is the largest and that of Co-doped TiO_2_ films is the smallest. Compared with the undoped TiO_2_ film, the addition of dopant decreases *A*_0_ and increases *Γ*, which suggests that the Urbach tail absorption characteristics were formed. Note that it is common to observe the development of an Urbach tail on doping transition metal oxides [45,46].

**Table 1:** **The fitted parameters of the TM-doped TiO**_**2 **_**films determined by the SE spectra**

		***Г *** **(eV)**	***E***_**OBG **_**(eV)**	***ϵ***_***∞***_	***A***_**0 **_**(eV**^**3/2**^**)**	**df (nm)**	**ds (nm)**	***C***_**TM **_**(%)**
Undoped		0.02 ± 0.01	3.58 ± 0.01	0.11 ± 0.03	136.6 ± 10	355 ± 10	5 ± 2	
Dopant content								
Fe	0.01	0.030 ± 0.01	3.56 ± 0.02	0.260 ± 0.02	132.31 ± 12	288 ± 8	3 ± 1	0.8
	0.03	0.085 ± 0.06	3.54 ± 0.02	0.087 ± 0.02	126.23 ± 20	265 ± 6	4 ± 2	2.7
Ni	0.01	0.035 ± 0.02	3.53 ± 0.01	0.1 ± 0.04	134.48 ± 13	233 ± 7	3 ± 1	0.9
	0.03	0.036 ± 0.03	3.50 ± 0.01	0.517 ± 0.11	128.18 ± 14	219 ± 6	3 ± 1	2.9
Co	0.01	0.042 ± 0.01	3.48 ± 0.02	0.528 ± 0.10	125.11 ± 11	215 ± 5	3 ± 2	0.8
	0.03	0.106 ± 0.04	3.43 ± 0.01	0.353 ± 0.15	118.9 ± 6	206 ± 5	4 ± 2	2.8

The film thickness (df), the thicknesses of the surface rough layer (ds), and the parameter value of Adachi's model (*A*_0_) for TM-doped TiO_2_ films with dopant content extracted from the simulation of SE in Figure 7. The 90% reliability of the fitted parameters is shown with ± sign. The TM atom composition *C*_TM_ derived by the XPS spectra is also listed.

**Figure 8:**
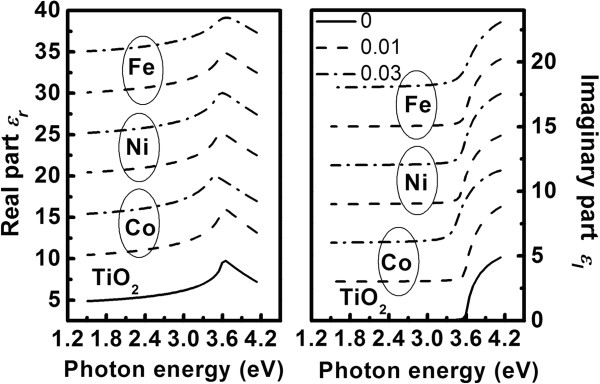
**Imaginary part *ϵ***_**i **_**and real part *ϵ***_**r **_**of the complex dielectric functions of the undoped and TM-doped TiO**_**2 **_**films.** For clarity, the *ϵ*_i_ and part *ϵ*_r_ of the films are shifted by 2 and 5, respectively.

Figure 8 depicts the variation in dielectric function of the TM-doped TiO_2_ films with photon energy. In general, in all samples, we found that the real part *ϵ*_r_ of the dielectric function increases and gradually nears the maximum, and then decreases due to the Van Hove singularities. This is the typical optical response of dielectric or semiconductor materials [44]. The imaginary part *ϵ*_i_ of the dielectric function nears zero in the transparent region (*E*_OBG_ > *E*) and sharply increases further with increasing photon energy in the absorption region (*E*_OBG_ < *E*).

The dopant content dependence of the *E*_OBG_ of the TM-doped TiO_2_ films is presented in Figure 6c. It is can be seen that the *E*_OBG_ of the TM-doped TiO_2_ films decreases with increasing dopant content. Note that at the same dopant content, the *E*_OBG_ value of the Co-doped TiO_2_ films is the smallest and that of the Fe-doped TiO_2_ films is the largest. By comparing the *E*_OBG_ value with the rutile content of the TM-doped TiO_2_ films (in Figure 6c), it is found that the change of *E*_OBG_ value is related to the fraction of rutile phase, except the doping. This suggests that *E*_OBG_ can be tuned not only by dopant type but also by dopant content. The undoped TiO_2_ film has little rutile phase detected by XRD, and the *E*_OBG_ value is about 3.58 ± 0.01 eV. For the *x* = 0.01 TM-doped TiO_2_ films, the rutile phase is minimal, and the *E*_OBG_ value is about 3.56 ± 0.02, 3.53 ± 0.01, and 3.48 ± 0.02 eV for Fe, Ni, and Co-doped TiO_2_ films, respectively. However, when dopant content reaches 0.03, the rutile phase is prominent for Co- and Ni-doped TiO_2_ films, and the *E*_OBG_ value is about 3.43 ± 0.01 and 3.50 ± 0.01 eV, respectively. For Fe-doped TiO_2_ film, the anatase phase is still prominent, and the *E*_OBG_ value is 3.54 ± 0.02 eV. These values of *E*_OBG_ for all samples are larger than those in the literatures [17,18,47] but near the reported values of rutile TiO_2_ films [44].

As shown in Figures 5 and 6c, the results indicate that the undoped TiO_2_ film is mainly composed of anatase phase and a minor rutile phase. Thus, the ARJs between the anatase and rutile phases are embedded within the anatase phase [15]. The electronic mobility from anatase-to-rutile phases is affected by the junctions. To some extent, the ARJ structure is electronically disordered. In addition, oxygen vacancies increase with increasing dopant content, which also results in the electronic disorder in the samples. Therefore, the increase of the disorder leads *E*_OBG_ to shift to lower energy [17,18,47]. With the same dopant content, the disorder in the Co-doped TiO_2_ films is the strongest and the *E*_OBG_ value is the smallest.

### Magnetic properties of the TM-doped TiO_2_ films

**Figure 9:**
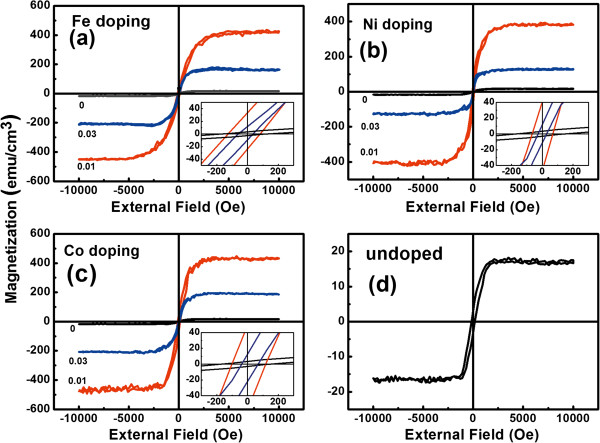
***M-H *curves of TM-doped TiO**_**2 **_**films. (a)** Fe doping. **(b)** Ni doping. **(c)** Co doping. **(d)** Undoped.

Magnetization (*M*) versus magnetic field (*H*) curves of TM-doped TiO_2_ films are displayed in Figure 9. The ferromagnetic hysteresis curves are clearly found for all samples, which indicate that the undoped and doped TiO_2_ films exhibit ferromagnetic behavior. The results are similar to those of the literature [21,48-51]. In addition, the *M* values of *x* = 0.01 Fe-, Ni-, and Co-doped TiO_2_ films at 10^4^ Oe were the largest and about 419.7, 386.5, and 445.6 emu/cm^3^, respectively. The *M* values of doped samples decrease with increasing metal element contents, which is similar to the Ni-doped TiO_2_ powders [21] and Fe-doped TiO_2_ films [52]. Generally, the magnetization of samples should increase with increasing magnetic ions, but the magnetic data of these samples do not support it. These magnetic phenomena are extraordinary and different from the magnetic results of the literature [7-11,21], which suggest that there are complex magnetisms in these samples.

The ferromagnetic properties of the anatase or rutile phase TM-doped TiO_2_ system have been widely investigated; however, there are few reports of magnetic change due to the phase fraction change. Recently, Bahadur et al. found that the magnetic moment of Ni-doped mixed crystalline TiO_2_ powders increases and then decreases with increasing Ni content [21]. They suggested that the observed ferromagnetic states may originate from the spin ordering through exchange interactions between the holes trapped in the oxygen 2*p* orbital adjacent the Ni site, which substitutes Ti sites. However, in their reports, rutile content decreases with increasing Ni content, indicating that their theory may not fit for our samples because the rutile content of the present doped TiO_2_ films increases. Additionally, Jiang et al. suggested that the decrease in the saturation magnetization may be related to the antiferromagnetic contribution with increasing dopant content in the Fe-doped TiO_2_ films [52]. Although their samples are mixed crystalline, the authors had not taken the ARJs into account.

It is known that TiO_2_ shows a strong polaronic effect in which the carrier effective mass becomes bigger due to strong electron–phonon interactions [53,54]. A polaronic electron will spend most of its time near an oxygen vacancy when it is trapped in the vacancy. Then the trapped electron can form an *F*-center. In the center, the trapped electron occupying an orbital effectively overlaps the *d* shells of the surrounding magnetic ions. Therefore, a possible origin of ferromagnetism is an *F*-center-bound magnetic polaron, which is formed by an electron trapped in an oxygen vacancy and its neighboring magnetic impurity ions [8,51]. In other words, the room-temperature ferromagnetism of TM-doped TiO_2_ films was induced mainly by the magnetic polarons formed by the localized electrons surrounded by magnetic impurities.

There are oxygen vacancies in our samples and the vacancies promote the ART. Thus, the magnetic properties of the samples may be related to the influence of the ART on the magnetic polarons. According to XRD analysis, the ART easily occurs in anatase TiO_2_ lattice with oxygen vacancies. The ARJs emerging during the course of ART will reduce the number of the trapped electrons. That is to say, these ARJs may destroy the magnetic polarons in anatase/rutile TiO_2_, which results in the decrease in magnetization. Of course, the magnetic mechanism of mixed crystal TM-doped TiO_2_ is an open issue and needs further study in depth.

## Conclusions

The TM-doped TiO_2_ films (TM = Co, Ni, and Fe) have been deposited on Si substrates by a sol–gel route. The additives promote the ART of the TiO_2_ films. The influence of Co, Ni, and Fe on the ART was compared. With the same dopant content, Co doping catalyzing the ART is more obvious than those of Ni doping and Fe doping, which is attributed to the different strain energy induced by oxygen vacancies and the difference in valence and ionic radii of Co^2+^, Ni^2+^, and Fe^3+^. The decreases of the *E*_OBG_ are related to the enhancement of disorders induced by the ARJs in the samples. The undoped TiO_2_ film exhibited weak magnetic properties, while ferromagnetic behaviors were clearly observed for TM-doped TiO_2_ films. The magnetizations of the TM-doped TiO_2_ films decrease with increasing dopant content, which may be related to magnetic polarons in the samples. The final explanation on their magnetic properties still remains a puzzle, and the true mechanism deserves further study.

## Competing interests

The authors declare that they have no competing interests.

## Authors’ contributions

JT carried out the preparation of sol–gel, participated in the data analysis, and drafted the manuscript. HG carried out the tackling SE and modified the manuscript. HK participated in the preparation of the samples. PY participated in the design of the study and performed the data analysis. JC and WZ conceived of the study and participated in its design and coordination. All authors read and approved the final manuscript.
